# Blockade of TNFα to Improve Human CD34+ Cell Repopulating Activity in Allogeneic Stem Cell Transplantation

**DOI:** 10.3389/fimmu.2018.03186

**Published:** 2019-01-22

**Authors:** Vitalyi Senyuk, Pritesh Patel, Nadim Mahmud, Damiano Rondelli

**Affiliations:** Division of Hematology/Oncology, Department of Medicine and UI Cancer Center, University of Illinois at Chicago, Chicago, IL, United States

**Keywords:** CD34, TNFα, transplantation, GVHD, T cells, repopulating activity

## Abstract

Early release of TNFα after hematopoietic stem cell transplantation (HSCT) correlates with development of acute graft-vs.-host disease (GVHD). Here we tested the effect of TNFα and alloreactive T cells on early hematopoietic HSC genotype and function. Addition of TNFα (10 ng/ml) in liquid cultures with CD34+ cells for 6–72 h resulted in the downregulation of genes associated with stem cell activity, such as DNMT3A, DNMT3B, TET1, TET2, SOX2, NANOG, and OCT4, whereas no significant effect was observed on DNMT1 and GATA2 expression. These findings were reversed by using an anti-TNFα antibody. Similar gene downregulation was observed when CD34+ cells were co-cultured with alloreactive T cells CD34+ cells for 48–72 h, and this effect was partially prevented by rapamycin and an anti-TNFα antibody. CD34+ cells pre-incubated with TNFα for 48 h and transplanted in irradiated NOD-SCID ɤ^null^ (NSG) mice showed a reduced myeloid engraftment compared to control mice. By using a xenograft model recently developed in our lab, we co-transplanted CD34+ cells and allogeneic T lymphocytes at 1:0.1 ratio in one group that also received etanercept (TNFα inhibitor) at 100 μg intra-peritoneum (i.p.) on days −1,+1,+3,+5 post-HSCT, and in the control group. At 6 weeks post-transplant, mice that received etanercept had a significantly higher number of marrow huCD45+CD34+CD38- early stem cells (*p* = 0.03) and a reduced number of huCD45+CD3+ splenic T cells (*p* = 0.04) compared to controls. The repopulating activity of marrow cells from mice treated with etanercept vs. controls was tested in secondary transplants. Although the overall engraftment was similar in the two groups, CD34+ cells isolated from recipients of marrow from the etanercept group showed a significantly greater expression of stem cell-associated genes and a higher number of CD45+CD34+CD38- cells than in controls (*p* = 0.03). Our findings suggest that early TNFα increase post-transplant can affect long-term stem cell engraftment, and that blockade of TNFα early after transplant may limit a cytokine-mediated suppressive effect on repopulating stem cell function.

## Introduction

The engraftment of donor hematopoietic stem cells (HSC) after transplantation requires a profound immunosuppression of the host to prevent the risk of rejection. However, the immunosuppression of the host also favors the expansion of donor immune cells that can target non-hematopoietic tissues and cause graft-vs.-host disease (GVHD) (1). The immunologic events determining GVHD start immediately after transplant and are largely based on an initial release of pro-inflammatory cytokines caused by the effect of chemotherapy/radiotherapy and antigen presenting cell: T cell interaction (1, 2). In fact, patients undergoing an allogeneic HSC transplant (HSCT) from HLA matched donors receive GVHD prophylaxis starting from the time of transplant and, in case of transplant from HLA haploidentical donors, with high doses of cyclophosphamide administered on days +3 and + 4 after transplant (3).

TNFα is a pro-inflammatory cytokine released particularly by donor T cells upon transplantation, and has previously been demonstrated to play a key role in the initial immune process leading to GVHD, both facilitating the activation of antigen presenting cells (APC) and the expansion of alloreactive T cells (4, 5). Moreover, detection of an increased serum level of TNFα receptor 1 in patients within the first week after transplantation is one of the biomarkers predicting future development of GVHD (6).

Previous findings from our lab demonstrated that TNFα mediates a direct effect of T cells on a subset of CD34+ cells hematopoietic progenitors, inducing their differentiation into the monocytic/dendritic lineage and increasing their direct as well as indirect antigen presenting function (7). Here we tested the question whether early hematopoietic stem cells with repopulating stem cell activity could also be a target of TNFα, thus hypothesizing that stem cell engraftment after transplantation could be affected by TNFα. We analyzed the *in vitro* effect of TNFα, as well as of allogeneic T cells, on CD34+ cell expression of genes regulating DNA methylation or pluripotency, such as DNMT1, DNMT3A, DNMT3B, NANOG, OCT4, SOX2 (8, 9). Then, we utilized a xenograft transplant (10) model to study the *in-vivo* effect of TNFα on HSC and the role of a TNFα inhibitor after co-transplantation of CD34+ and allogeneic T cells. The results shown here suggest that TNFα can affect early HSC and that blockade of TNFα may preserve a pool of stem cells with repopulating activity. Based on these findings, new therapeutic strategies may be tested to better protect stem cell engraftment after allogeneic transplantation.

## Materials and Methods

### Cell Separation

Healthy donor G-CSF mobilized peripheral blood stem cells (PBSC) from AllCells (Alameda, CA) and PB cells from healthy volunteers were utilized in this study. Mononuclear cells (MNC), CD34^+^ cells and CD3+ T cells were purified as previously described (10). Isolated CD34+, or T cell samples were acquired on a FACS Calibur^TM^ (Becton Dickinson) and analyzed using the Cell Quest ^TM^ software (Becton Dickinson), and showed, on average, >95% cell purity.

### Flow Cytometry

Fluorescein isthiocyanate (FITC), or phycoerythrin (PE), or peridin chlorophyll protein (PerCP), conjugated mAbs (CD45, CD34, CD38, CD33, CD3) or isotype controls (Becton-Dickinson, San Jose', CA) were employed. Stained cells were washed twice in PBS and sample acquisition and analysis was performed within 2 h on a FACSCaliburTM (Becton Dickinson).

### Co-cultures of CD34+ and T Cells

Purified human CD34+ cells (1–2 x 10^5^ cells) were co-cultured with human allogeneic T cells at 1:0.1, or 1:2 ratio in round-bottomed 96-well plates for 48–72 h at 37°C in a 5% CO2 humidified atmosphere, as previously described. In selected experiments, CD34+ cells and T cells were cultured in the presence of the following molecules described: TNFα, Rapamycin, Cyclosporin A (Sigma-Aldrich (St. Louis, MO), Mycophenolate Motefil (Cayman Chemical Company, Ann Arbor, MI), Abatacept (Bristol Meyers Squibb, New York, NY), rabbit anti-thymocyte globulin (rATG, Thymoglobulin, Genzyme, Cambridge, MA), anti-TNFα antibody (AF-210-NA) from R&D Systems (Minneapolis, MN).

### qRT-PCR

CD34+ cells re-isolated on human CD34+ MicroBead Kit UltraPure (Miltenyi Biotec, Bergisch Gladbach, Germany) after MLC or after transplantation were used for total RNA extraction with TRIzol reagent (Life Technologies Corporation, Grand Island, NY). RNA was transcribed into cDNA with SuperScript^®^ III First-Strand Synthesis SuperMix (Life Technologies Corporation, Grand Island, NY) and analyzed with SYBR green (Applied Biosystems, Inc., Grand Island, NY) on the 7500 FAST Real Time PCR detection system (Applied Biosystems, Inc., Grand Island, NY). The human primers used are: ACTB, forward: 5-ggacttcgagcaagagatgg-3′, reverse: 5′-agcactcgtgttggcgtacag-3′; DNMT1, forward: 5′-tgctgaagcctccgagat-3′, reverse: 5′-ttctgttaagctgtctctttcca-3′; DNMT3A, forward: 5′-tacttccagagcttcagggc-3′, reverse: 5′-attccttctcacaacccgc-3; DNMT3B, forward: 5′-gagattcgcgagcccag-3′, reverse: 5′-tctccattgagatgcctggt-3′; TET1, forward: 5′-gagggaaaagaagcccaaag-3′, reverse: 5′-tcttccccatgaccacatct-3′; TET2, forward: 5′-agaaaagggaaaggagagcg-3′, reverse: 5′-gagagggtgtgctgctgaat-3; TET3, forward: 5′-gccggtcaatggtgctagag-3′, reverse: 5′-cggttgaaggtttcatagagcc-3′; NANOG, forward: 5′-gatttgtgggcctgaagaaa-3′, reverse: 5′-cagggctgtcctgaataagc-3′; OCT4, forward: 5′-gtggaggaagctgacaacaa-3′, reverse: 5′-ggttctcgatactggttcgc-3; SOX2, forward: 5′-aaccccaagatgcaccaactc-3′, reverse: 5′-gcttagcctcgtcgatgaac-3,. GATA2, forward: 5′- cacaagatgaatgggcagaa−3′, reverse: 5′- acaatttgcacaacaggtgc−3′.

### TNFα Blockade

*In vitro* TNFα blockade was tested in MLC assays with anti-TNFα antibody (AF-210-NA). In titration experiment, we tested 0.1 μg/ml, 0.5 μg/ml and 1 μg/ml of anti-TNFα antibody, and in selected experiments at 5 μg/ml. The tested anti-TNFα/ TNFα excess range (10x−100x) covers whole possible TNFα neutralization range, according to the manufacturer's guide. *In vivo* TNFα blockade was tested in NSG mice co-transplanted with CD34+ and allogeneic T cells at 1:0.1 ratio by injecting etanercept (Enbrel, Immunex Corporation, Thousand Oaks, CA) intra-peritoneum (i.p.).

### Transplantation

Immunodeficient nonobese diabetic/*ltsz-scid*/*scid* (NOD/SCID) IL2 receptor gamma chain knockout mice (NSG) were purchased from the Jackson Laboratories (Bar Harbor, ME) and housed in a strict barrier environment. The study was approved by UIC Animal Care Committee and performed in accordance with national guidelines of laboratory animal care. Human CD34+ cells were initially incubated with or without TNFα (10 ng/ml) *in-vitro* for 48 h, re-isolated immune-magnetically using a human CD34+ MicroBead Kit UltraPure (Miltenyi Biotec, Bergisch Gladbach, Germany) and 2 × 10^5^ cells were transplanted intravenously (i.v.) into sublethally irradiated (300 cGy) NSG mice as previously described (10). In a second set of experiments, purified CD34+ cells (2 × 10^5^/mouse) were mixed at 1:0.1 ratio with allogeneic T cells and then injected i.v. into sublethally irradiated (300 cGy) NSG mice. A group of mice was also injected intra-peritoneum (i.p.) with etanercept at 100 μg/mouse on days: −1, +1, +3 and +5 post-transplant (*n* = 10 mice/group). At day 42 post-transplant, marrow cells were collected to analyze engraftment, as well as to perform secondary transplants. To this purpose, marrow cells obtained after primary transplant were depleted of human T cells on a Miltenyi column after incubating the cells with MicroBeads conjugated to monoclonal anti-human CD3 antibody (Miltenyi Biotec, Bergisch Gladbach, Germany). Then secondary NSG mice were transplanted with 5 × 10^6^ T cell depleted marrow cells/mouse from the etanercept and control groups (*n* = 5 mice/group). Forty-two days following second transplant, mice were sacrificed and analyzed for stem cell engraftment in the marrow andspleen, as previously described (10). Human cell engraftment was assessed by measuring the expression of huCD45 marker. Analysis of all markers on marrow or spleen cells was performed on gated huCD45+ cells.

### Statistical Analysis

Student *t*-test was performed to compare 2 series of data. Statistical tests were performed by using Graph Pad Prism version 7.0 (GraphPad Inc., San Diego, CA).

## Results

### TNFα-mediated Downregulation of Early Stem Cell Gene Expression in CD34+ Cells

**Figure 1:**
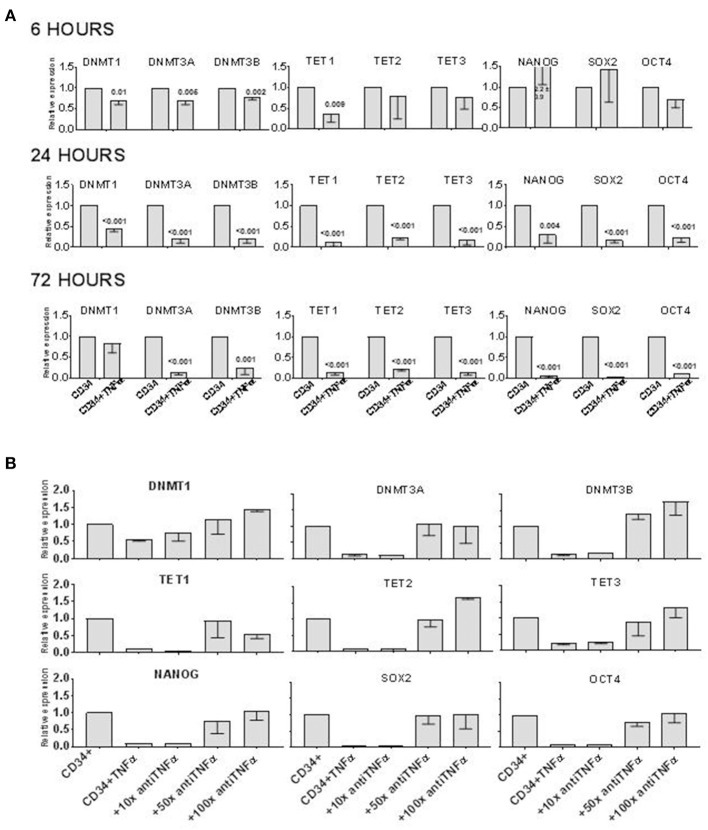
TNFα downregulates epigenetics- and pluripotency-related genes in CD34+ cells *in vitro*. **(A)** CD34+ cells were incubated *in vitro* ± TNFα (10 ng/ml) for 6–72 h, before analyzing expression of TET1, TET2, TET3, DNMT1, DNMT3A, DBMT3B, NANOG, SOX2, OCT4 genes by qRT PCR. Gene expression in CD34+ cells alone was arbitrary taken as 1 for each gene. ACTB expression was used as normalization control. The data is shown as mean values of 4 separate experiments and standard deviation bars are included. Differences in the expression of each gene in cells treated with TNFα and controls were calculated by *t*-test and *p*-values are shown. **(B)** The effect of TNFα on gene expression in CD34+ cells was prevented by adding an anti-TNFα antibody to liquid culture with CD34+ cells. The antibody (1:100 = 1 μg/ml) was added at 10x, or 50x, or 100x excess of TNFα (by mass) to CD34+ cells cultured alone or with TNFα for 72 h before qRT PCR analysis. Gene expression in CD34+ cells alone was arbitrary taken as 1 for each gene. ACTB expression was used as normalization control. The data is shown as mean values of 4 separate experiments and standard deviation bars are included.

It has been previously demonstrated that TNFα induces the differentiation of a subset of CD34+ cells committed to the monocytic-dendritic lineage within 3–5 days of liquid culture (7). To test whether also early hematopoietic progenitors can be targeted by the effect of TNFα, we incubated human CD34+ cells with or without TNFα at 10 ng/ml for 6–72 h and then extracted their mRNA to measure the expression of genes associated with self-renewal or pluripotent stem cell activity, such as: GATA2, DNMT1, DNMT3A, DNMT3B, TET1, TET2, TET3, NANOG, SOX2, and OCT4 (Figure 1A). In 5 separate experiments, GATA2 expression was not affected by TNFα at any time point (data not shown). On the contrary, all the other genes were significantly downregulated at either one or all three time points, compared to control experiments without TNFα. The low expression of DNMT3A, DNMT3B, TET2, NANOG, SOX2, and OCT4 at 24 and 72 h suggested that genes regulating stem cell proliferation can be rapidly downregulated by TNFα in CD34+ cells. We then incubated the CD34+ cells with TNFα and increasing doses of a blocking anti-TNFα antibody for 72 h. The expression of all the genes previously downregulated was restored to the level of control CD34+ in the presence of higher doses of the blocking antibody (Figure 1B). In control experiments, the anti-TNFa antibody alone did not affect gene expression on CD34+ cells (data unshown). These findings showed that TNFα directly modified the expression of multiple genes in CD34+ cells.

### T Cell-mediated Downregulation of Early Stem Cell Gene Expression in CD34+ Cells

**Figure 2:**
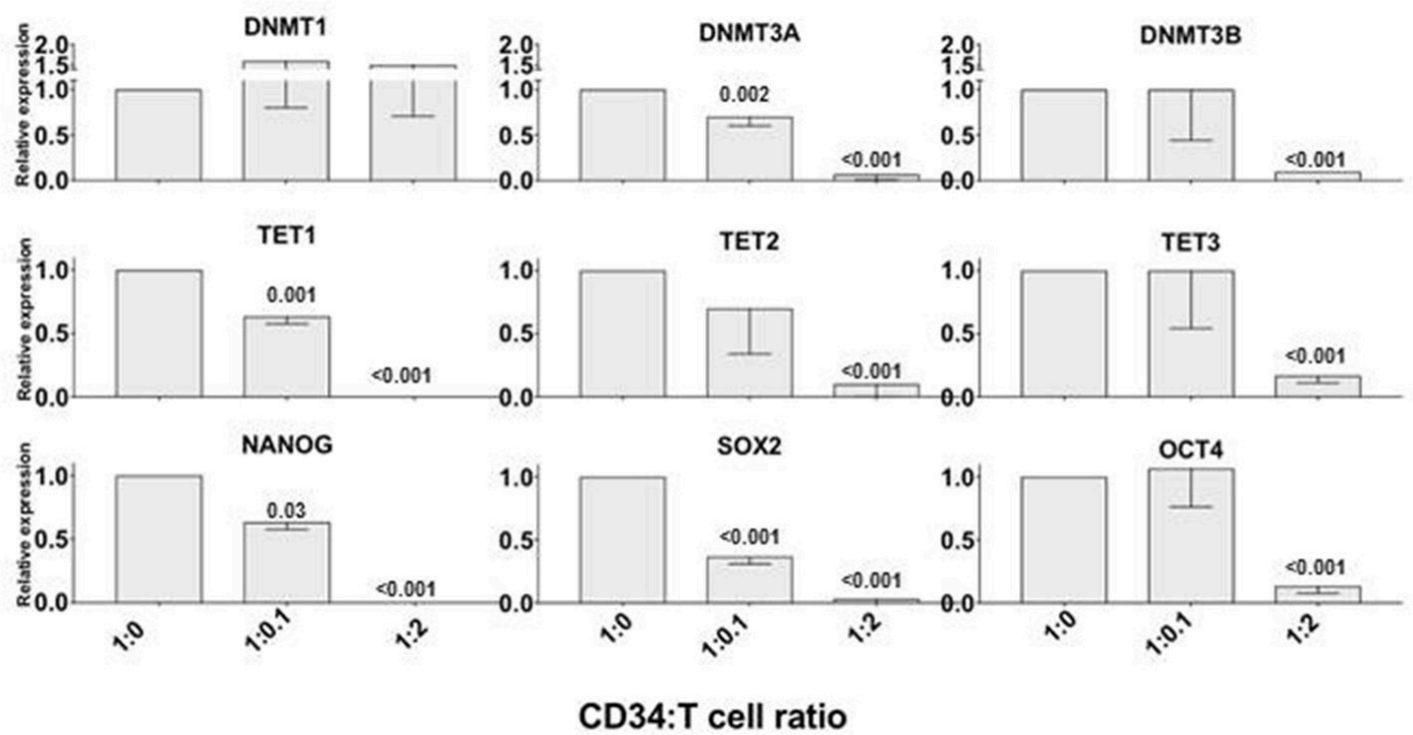
Alloreactive T cells rapidly downregulate epigenetics- and pluripotency-related genes in CD34+ cells. Purified CD34+ cells were co-cultured with allogeneic CD3+ T cells at 1:0 (control), 1:0.1 and 1:2 ratios for 72 h; then were re-isolated immunomagnetically before analyzing the expression of TET1, TET2, TET3, DNMT1, DNMT3A, DBMT3B, NANOG, SOX2, OCT4 genes by qRT PCR. Gene expression in CD34+ cells cultured without T cells was arbitrary taken as 1 for each gene. ACTB expression was used as normalization control. The data is shown as mean values of 3 separate experiments and standard deviation bars are included. Differences in the expression of each gene in CD34+ cells co-cultured with T cells vs. control were calculated by *t*-test and significant *p*-values are shown.

We previously demonstrated that allogeneic T cells induce a rapid differentiation of a subset of CD34+ cells into monocytic/dentritic cells, mostly mediated by TNFα (7). Based on the observation above that TNFα downregulated the expression of genes associated with DNA methylation and pluripotent stem cell activity, we tested whether alloreactive T cells can induce a similar effect. We incubated CD34+ cells with allogeneic CD3+ T cells at 1:0 (control), 1:0.1 or 1:2 ratio as previously described (10). After 72 h, CD34+ cells were immunomagnetically re-purified and their mRNA was isolated to assess the gene expression. In the presence of low number of T cells (1:0.1 ratio), only DNMT3A, TET1, and NANOG expression was decreased, whereas at higher concentration of T cells (1:2 ratio) all the genes tested were downregulated with the exception of DNMT1 (Figure 2) and GATA2 (not shown). These findings suggested that alloreactive T cells may affect the expression of some of the genes associated with early stem cell activity in CD34+ cells.

### Variable Effect of Standard Immunosuppressive Agents on T Cell-mediated Epigenetic Changes in CD34+ Cells

**Figure 3:**
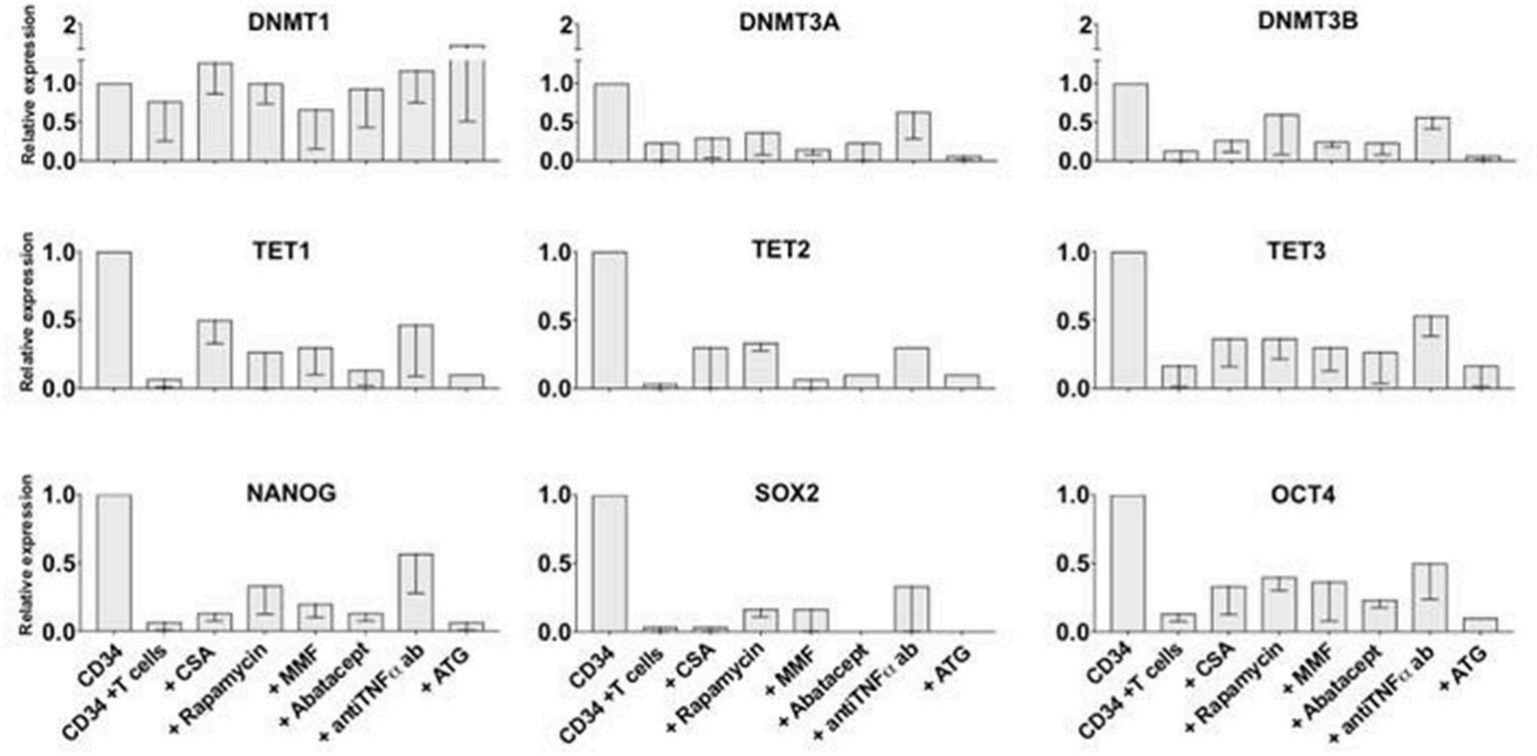
Partial effect of standard immunosuppressive agents in preventing T cell-mediated epigenetic and pluripotency gene downregulation in CD34+ cells *in-vitro*. CD34+ and allogeneic T cells were co-cultured at 1:2 ratio for 72 h with or without one of the following standard immunosuppressive molecules: cyclosporin A, rapamycin, mycophenolate mofetil, abatacept, anti-TNFα antibody, ATG. CD34+ cells were then re-isolated immunomagnetically and analyzed by qRT PCR. Gene expression in CD34+ cells cultured without T cells as control was arbitrary taken as 1 for each gene. ACTB expression was used as normalization control. The data is shown as mean values of 3 separate experiments and standard deviation bars are included.

Since we observed that alloreactive T cells rapidly affect the expression of genes that could regulate stem cell long term engraftment, we investigated on whether an anti-TNFα antibody or immunosuppressive molecules commonly used as GVHD prophylaxis could prevent T cell effect on CD34+ cells. We performed 72 h liquid cultures with CD34+ and allogeneic T cells at 1:2 ratio or CD34+ alone, then we added standard immunosuppressive agents such as cyclosporine A (1 μg/ml), rapamycin (1 μg /ml), mycophenolate mofetil (0.5 μg/ml), abatacept (100 μg /ml), thymoglobulin (100 μg /ml) or an anti-TNFα antibody (5 μg/ml) (Figure 3). After 72 h the CD34+ cells were immunomagnetically re-isolated in order to extract the mRNA and test gene expression. Although none of the immunosuppressive drugs, or the anti-TNFα antibody, could completely prevent the T cell-mediated gene downregulation in CD34+ cells, a trend for a greater activity in preserving epigenetic and pluripotency gene expression was observed with rapamycin and anti-TNFα antibody, suggesting possible combination studies.

### Short Exposure to TNFα Reduces CD34+ Cell Engraftment After Transplant

**Figure 4:**
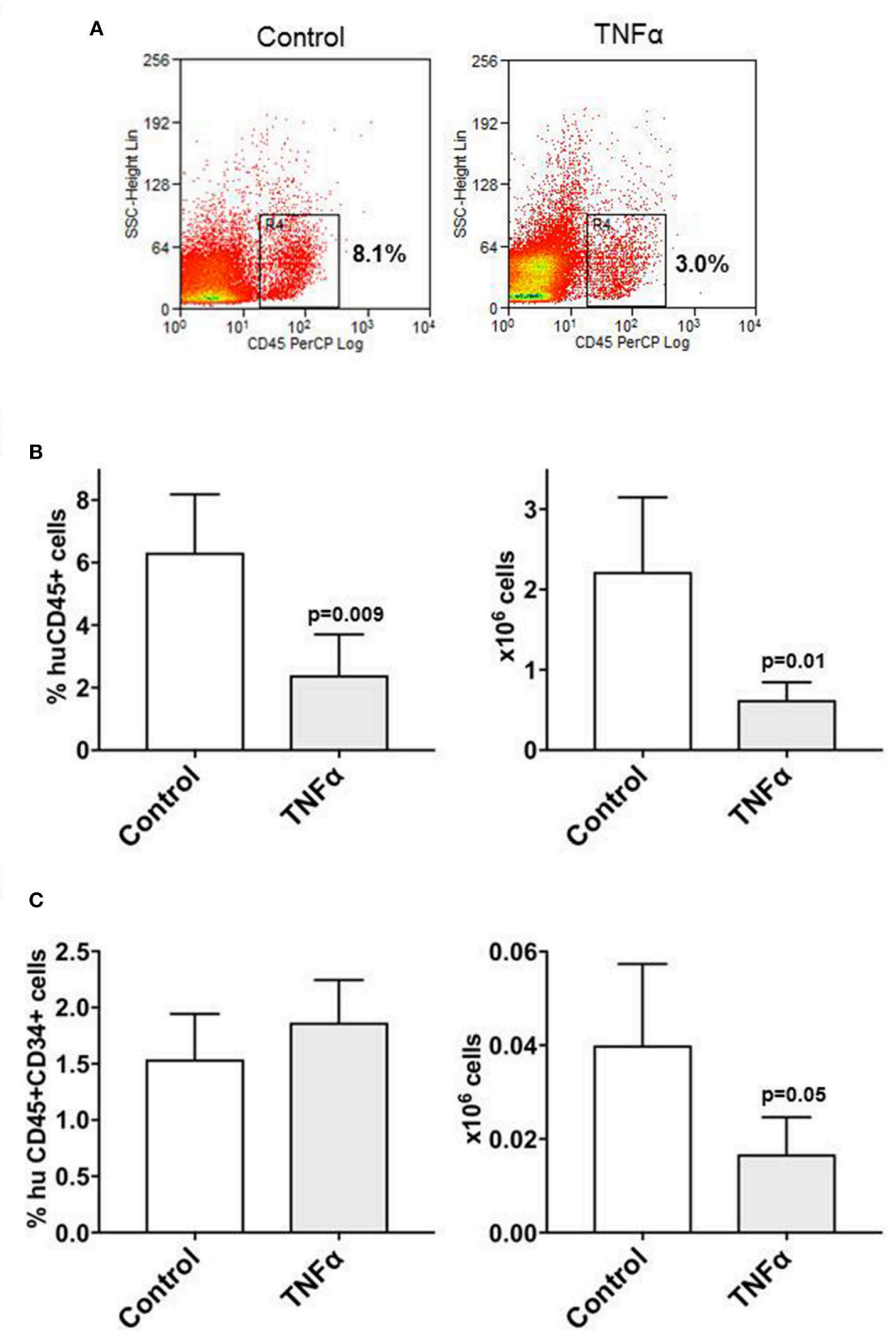
Pre-exposure of CD34+ cells to TNFα reduces stem cell engraftment in NSG mice. Purified CD34+ cells were initially cultured *in-vitro* with/without TNFα (10 ng/ml) for 48 h and then 2 sets of sublethally irradiated NSG mice (*n* = 5 each) were transplanted. Five weeks transplantation, the mice were sacrificed and cells isolated from the bone marrow were stained with anti-huCD45 and anti-CD34 antibodies. The reduced engraftment of human CD45+ cells in mice transplanted with CD34+ cells pre-treated with TNFα is shown in one representative example of cytofluorimetric analysis from each group of mice **(A)**. After transplant, the average percentage and absolute number of huCD45+ cells detected in the marrow of mice pre-treated with TNFα were significantly lower than in control animals **(B)**, as well as the absolute number of marrow huCD45+CD34+ cells **(C)**. Differences between groups were analyzed by *t*-test and significant *p*-values are shown.

In order to test whether TNFα may affect the repopulating activity of CD34+ cells, initial experiments were designed to transplant CD34+ cells into NSG mice after short exposure to TNFα. Liquid cultures of CD34+ cells with or without TNFα at 10 ng/ml concentration were carried out for 48 h. Analysis of gene expression (not shown) confirmed downregulation of genes associated with early stem cell activity, as described above. CD34+ cells pretreated with TNFα for 48 h, or untreated CD34+ cells as control, were washed and then transplanted into sublethally irradiated NSG mice at 1 × 10^5^ CD34+ cells/mice (*n* = 5 mice per group). Five to 6 weeks after transplant the mice were sacrificed and stem cell engraftment was measured in the marrow and spleen by flow cytometry. Mice transplanted with CD34+ cells pretreated with TNFα had a lower bone marrow stem cell engraftment compared to controls, shown in a representative example in Figure 4A, and documented as lower percentage and absolute number of huCD45+ cells (Figure 4B). We also analyzed whether exposure of CD34+ cells to TNFα would affect the pool of marrow CD34+ cells after transplant, possibly responsible for long term engraftment. The absolute number of CD34+ cells was found significantly lower in the marrow of mice transplanted with TNFα-pretreated CD34+ cells (Figure 4C). These findings are consistent with the hypothesis that a brief exposure to TNFα may impair the engraftment ability of CD34+ cells.

### Etanercept Preserves the Engraftment of CD34+CD38- Cells After Co-transplantation With Allogeneic T Cells

**Figure 5:**
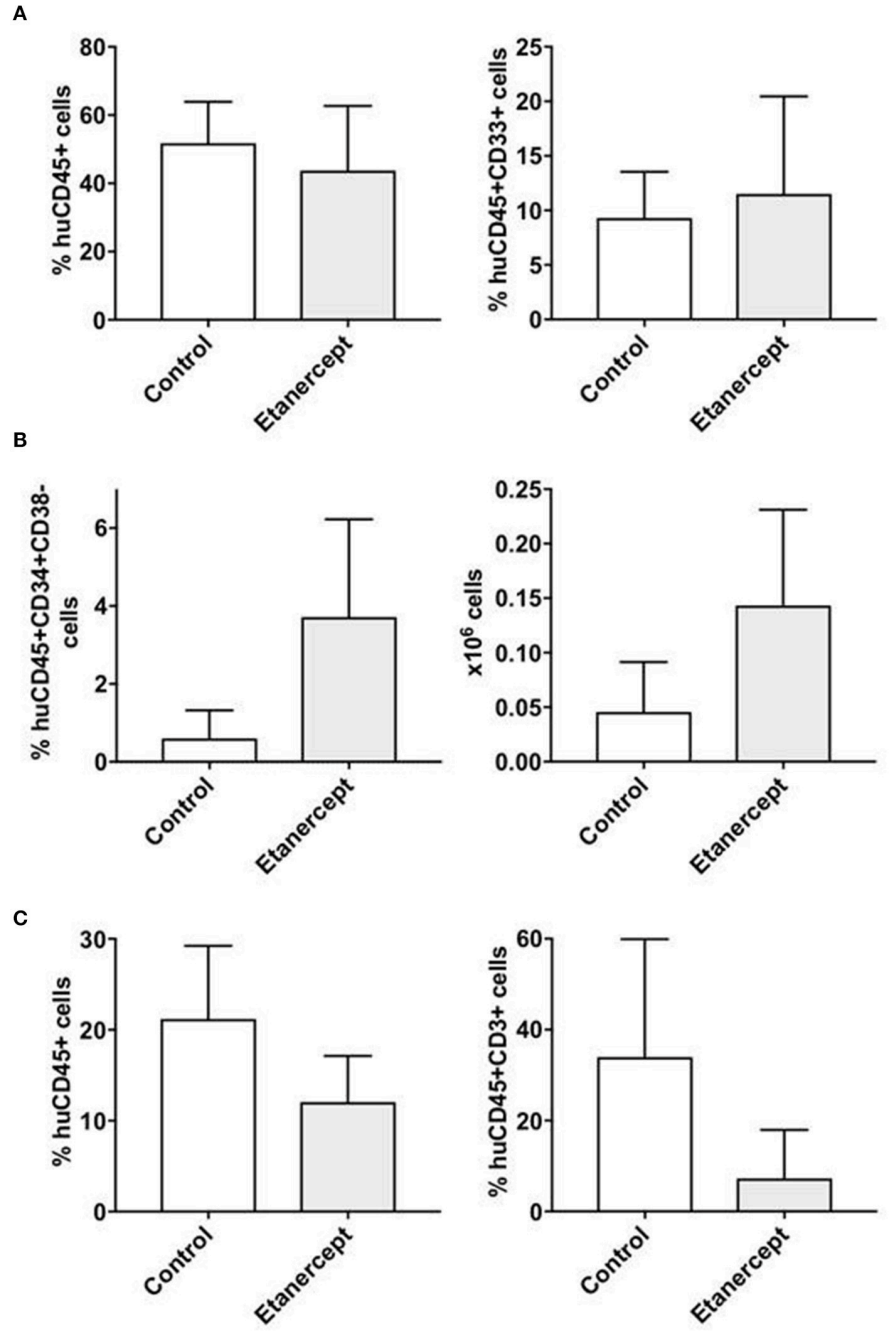
Peri-transplant treatment with etanercept improves the persistence of CD34+CD38- early hematopoietic progenitors after co-transplantation of CD34+ and allogeneic T cells in NSG mice. NSG mice were sublethaly irradiated and co-transplanted with CD34+ cells (2 × 10^5^/mouse) and allogeneic T cells at 1:0.1 ratio. One group of mice (*n* = 10) were also injected i.p. with TNFα inhibitor etanercept on days: −1, +1, +3 and +5 following transplantation, while the control group (*n* = 10) did not receive etanercept. Six weeks after transplantation, the mice were sacrificed and bone marrow and spleen cells were isolated and analyzed by flow cytometry to detect the percentage and the absolute number of human **(A)** myeloid (CD45+CD33+) and **(B)** lymphoid T (CD45+CD3+) cells, as well as for **(C)** early hematopoietic progenitors (CD45+CD34+CD38-) cells. The results are represented as mean values and standard deviation bars are shown. Differences between groups were analyzed by *t*-test and significant *p*-values are shown.

**Figure 6:**
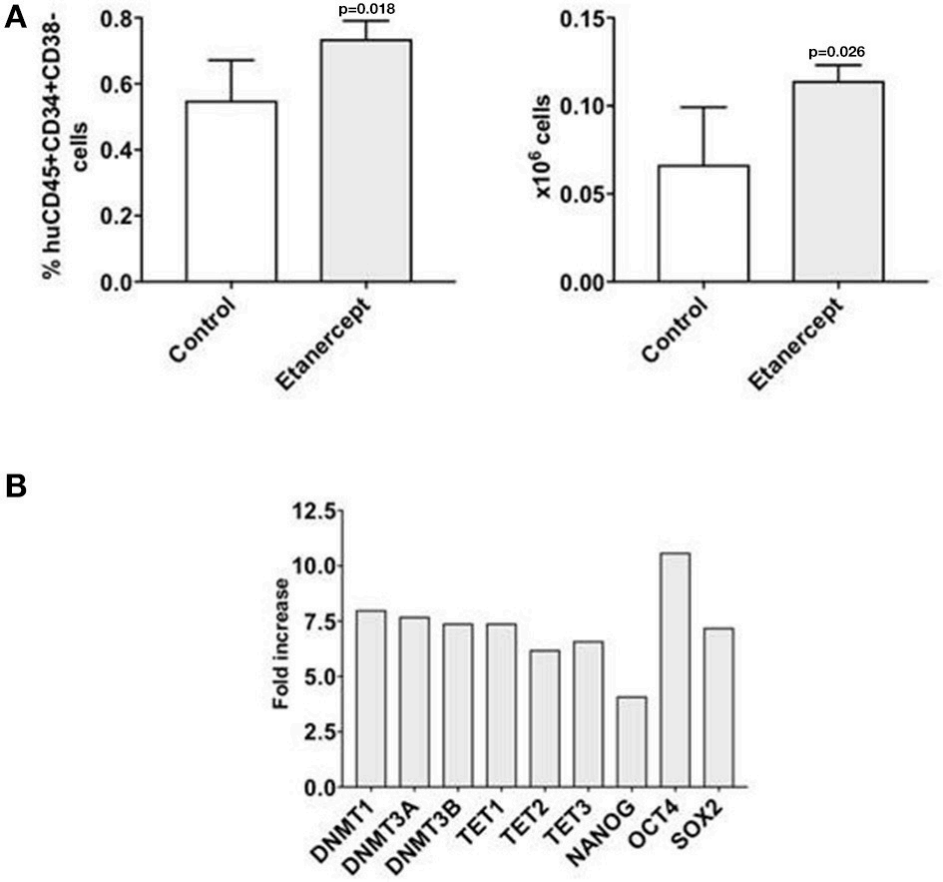
Early treatment with etanercept in primary transplant preserves the pool of CD34+CD38- hematopoietic progenitors after secondary transplant. Bone marrow cells were isolated from NSG mice 42 days after primary transplant with CD34+ and allogeneic T cells at 1:0.1 ratio, with/without treatment with etanercept. A fixed amount of 5 × 10^6^ T cell depleted marrow cells/mouse were re-transplanted in a secondary transplant in new NSG mice. After 42 more days, mice were sacrificed and bone marrow were isolated and analyzed by flow cytometry **(A)** to detect the percentage and the absolute number of human early hematopoietic progenitors (CD45+CD34+CD38-) cells. The results are represented as mean values and standard deviation bars are shown. Differences between groups were analyzed by *t*-test and significant *p*-values are shown. Another fraction of marrow cells were utilized to analyze the gene expression on CD34+ cells **(B)**. Because of the limited number of CD34+ cells, marrow cells from each group had to be pooled together to be re-isolated and analyzed by qRT PCR. In this experiment CD34+ cells from controls (untreated animals) were arbitrary taken as 1 for each gene. ACTB expression was used as normalization control. The results are shown as fold-increase of expression for each gene analyzed in the CD34+ cells isolated after secondary transplant in the etanercept group vs. control.

We recently observed that co-transplantation of CD34+ and allogeneic T cells at 1:0.1 ratio in a xenograft model results in low stem cell engraftment and in the expansion of T cells (10). Here we adopted this model to test whether *in-vivo* blockade of TNFα would improve the engraftment of CD34+ cells by reducing the effect of alloreactivity. Two groups of NSG mice were co-transplanted with CD34+ cells and T lymphocytes at 1:0.1 ratio and one of these groups was injected i.p. with etanercept at 100 μg on days −1, +1, +3 and + 5 post transplant. Six weeks after transplant, the marrow of mice treated with etanercept and those in the control group showed similar percentages of huCD45+ cells and human myeloid CD33+ cells (Figure 5A). Instead, the percentage and absolute number of early hematopoietic progenitors identified as CD45+CD34+CD38- cells were significantly higher in the etanercept group (*p* = 0.03), compared to control mice (Figure 5B). In addition, mice treated with etanercept showed a significantly lower expansion of T cells in the spleen, as demonstrated by a lower percentage of CD45+ and CD45+CD3+ cells in the spleen (Figure 5C). These findings suggested that early blockade of TNFα after transplant may preserve the pool of early hematopoietic stem cells. To test whether these cells also had a greater repopulating stem cell activity, a secondary transplant with marrow cells obtained from the etanercept and control groups was performed. Secondary NSG mice were transplanted with equivalent number of marrow cells collected from primary transplants and 6 week later (total 12 weeks) the marrow was analyzed for myeloid engraftment and persistence of early progenitor cells. Recipients of marrow cells from the etanercept and control groups had comparable percentages of CD45+ and CD45+CD33+ myeloid cells after secondary transplant (not shown). However, a higher percentage and absolute number of CD34+CD38- cells were detected in recipients of marrow cells from the etanercept group (Figure 6A). Finally, CD34+ cells from both the etanercept and control groups were re-isolated after transplant. Because of the small absolute number of CD34+ cells, we could not analyze each single mouse separately and marrows from either group were pooled before CD34+ cell selection and RNA extraction. The results of this experiment (Figure 6B) showed a 4–8 fold increase of gene expression in the etanercept group compared to mice that had not received etanercept not during primary transplant. Persistence of high expression of pluripotent genes in CD34+ cells from the etanercept group after secondary transplant did not correlate with a skiewed differentiation capacity since myeloid engraftment was comparable in the two groups. These data are consistent with initial hypothesis that release of TNFα immediately after transplant may affect long-term reconstitution of hematopoietic progenitors.

## Discussion

This study demonstrates that TNFα and alloreactive T cells rapidly affect human early hematopoietic precursors by downregulating the expression of genes associated with self-renewal and pluripotent stem cell activity, and affecting the engraftment of repopulating HSC *in-vivo*. A protective effect on repopulating HSC was elicited by blockade of TNFα in a xenograft model of co-transplantation of CD34+ and allogeneic T cells.

The role of TNFα in the development of GVHD has been extensively studied both in experimental models and in the clinical setting (1, 2, 4, 11–13). In this latter, it was also demonstrated that increased serum levels of TNFR1 on day 7 after transplant were shown to predict patients who then developed GVHD (6, 14–16). TNFα receptors (TNFR1 and TNFR2) were previously detected also on CD34+ hematopoietic progenitors (17, 18). Stimulation of TNF receptors with TNFα was then shown to upregulate the expression of interferon ɤ (IFNɤR) and FasL receptors, thus increasing their susceptibility to inhibitory effects of TNFα or IFNɤ. However, although some experimental models suggested a negative effect of TNFα in regulating normal hematopoiesis *in-vivo* (19, 20), other studies observed an impaired long-term hematopoietic reconstitution in mice lacking TNFR1 (21). We previously showed that committed CD34+ progenitors can stimulate allogeneic T cell responses *in-vitro* (22, 23) via B7:CD28 costimulation, and that ~70% of PB CD34+ cells upregulated CD40 costimulatory molecule and increased their immunostimulatory activity following 24 h exposure to TNFα (24). Endogenous TNFα released in co-cultures of CD34+ and allogeneic T cells was then demonstrated to mediate the rapid differentiation of a subset of CD34+ cells into monocytic/dendritic cells, whereas co-transplantation of CD34+ and T cells into NOD/SCID mice resulted in increased differentiation of mature dendritic cells (7). Our current study shows for the first time that within 6–72 h in liquid culture, TNFα downregulated the expression of some of the genes regulating stem cell methylation, such as DNMT3A/B, or TET1, TET2, TET3, and other transcription factor genes, such as NANOG, SOX2, or OCT4 that are associated with pluripotent status of stem cells (8, 9). Since it has previously reported that: *de novo* DNA methylation through DNMT3A/B activity is essential for stem cell long term reconstitution activity (25, 26); concomitant reduction of DNMT3A and TET-mediated DNA methylation can impair both self-renewal and differentiation of hematopoietic stem cells (27); and transcription factors such as OCT4, SOX2, and NANOG contribute to maintaining the pluripotency properties of early stem cells (9), it is conceivable that the effect of TNFα may target the epigenetic regulation of early CD34+ cells capable of post-transplant long-term hematopoietic reconstitution. Based on these observations on the downregulation of some of the genes affecting DNA methylation concomitantly with a reduced engraftment ability of HSC, new studies can be started to address the causality of each gene dysregulation and CD34+ cell post-transplant engraftment capacity. Because the same genes downregulated by TNFα were also rapidly downregulated *in-vitro* by allogeneic T cells, the cytokine storm caused by alloreactive T cells in the early phase post-transplant may affect CD34+ cell function either through TNFα alone, or through a combination of multiple soluble factors. Indeed, the T cell mediated epigenetic changes on CD34+ cells were only partially prevented by an anti-TNFα antibody. Interestingly, when we tested the effect of immunosuppressive molecules that are commonly used to prevent GVHD, such as cyclosporine A, rapamycin, mycophenolate mofetil, thymoglobulin, abatacept, or an anti-TNFα antibody in co-cultures of CD34+ and allogeneic T cells, none of them could completely prevent gene downregulation, and only rapamycin and anti-TNFα ab showed some partial protective effect. Future studies will test the effect of combination of these two agents. Consistent with *in-vitro* data, transplantation of CD34+ cells exposed to TNFα for 48 h resulted in a lower engraftment. In addition, by utilizing a xenograft model of co-transplantation of CD34+ and allogeneic T cells recently developed in our lab (10), we showed that injection of etanercept in the first week after transplant maintained a greater pool of CD34+CD38- hematopoietic progenitors after both primary and secondary transplant. These findings are consistent with a recent study where the authors transplanted high numbers of human umbilical cord (UC) cells into NSG mice and assessed the engraftment after only 4 weeks, because of xenogeneic GVHD limiting mice survival. In this study, they demonstrated that UC T cells produced high levels of multiple cytokines, including TNFα, which directly impaired stem cell engraftment (28). Although they did not test the repopulating stem cell activity in secondary transplants, they clearly showed a negative effect of T cell-derived TNFα on short and long-term hematopoietic stem cell subsets by inducing stem cell apoptosis, and proved that stem cell engraftment in mice transplanted with UC grafts plus etanercept was comparable to those receiving T cell-depleted grafts. Similarly, we demonstrated that in mice transplanted with CD34+ and allogeneic T cells TNFα blockade with etanercept in the first week of transplant allowed a greater engraftment of CD34+CD38- cells, not only in primary but also in secondary transplants. This could be due to limiting T cell expansion and preventing a TNFα-mediated epigenetic dysregulation of the physiologic balance of stem cell self-renewal and differentiation activity of CD34+ cells. This hypothesis was indirectly supported by our observation of higher expression of DNMTs, TETs and pluripotency transcription factor genes in CD34+ cells obtained from the etanercept group after secondary transplants.

All these findings expand the knowledge of an early effect of TNFα on the human hematopoietic system after allogeneic stem cell transplantation. In a clinical setting, it is conceivable that the pool of newly transplanted stem cells may be partially affected by the cytokines released by alloreactive T cells. Depending on the overall number of stem cells transplanted into a patient, a small loss of stem cell function may not be clinically relevant since the graft may have an abundant number of residual stem cells to guarantee the engraftment. However, patients fully engrafted with donor cells and yet experiencing late post-transplant cytopenias (29), or those with cytopenias in the context of GVHD, could be affected by a T cell-mediated anti-stem cell reactivity. This could be identified as a cytokine-mediated graft-vs.-marrow disease. TNFα blockade was previously proven to ameliorate acute GVHD in combination with other immunosuppressive drugs (30–32). Our findings could prompt new studies testing etanercept in the context of GVHD prophylaxis, not only to better prevent GVHD, but also to protect donor HSC from the detrimental effect of TNFα and facilitate long-term engraftment.

## Author Contributions

VS, PP, and DR contributed to the study design. VS, NM, and PP performed all the experiments, VS and DR analyzed the data. VS and DR wrote of the manuscript. All the authors assisted in the critical review of the manuscript and approved the final version of the manuscript for submission.

### Conflict of Interest Statement

The authors declare that the research was conducted in the absence of any commercial or financial relationships that could be construed as a potential conflict of interest.
